# Integrated characterization of distinct immune subtypes linked to drug responsiveness in breast cancer

**DOI:** 10.3389/fimmu.2026.1795917

**Published:** 2026-05-05

**Authors:** Min Liu, Meina Yang, Bo-shi Fu

**Affiliations:** 1 Department of Pharmacy, The First Affiliated Hospital of Xi’an Jiaotong University, Xi’an, China; 2 Department of Pharmacology, School of Pharmacy, China Medical University, Shenyang, China; 3 Liaoning Key Laboratory of Molecular Targeted Anti-Tumor Drug Development and Evaluation, Liaoning Cancer Immune Peptide Drug Engineering Technology Research Center, Shenyang, China

**Keywords:** breast cancer, immune subtype, immunologic gene set, immunotherapy, prognosis, tumor immune microenvironment

## Abstract

**Introduction:**

Precision medicine emphasizes accurate patient stratification. However, existing molecular frameworks often fail to fully capture the intrinsic heterogeneity of breast cancer (BRCA). This study aims to establish a novel immune-based classification system using immunological gene sets to elucidate tumor heterogeneity and identify predictive biomarkers for optimizing clinical management.

**Methods:**

Non-negative Matrix Factorization (NMF) analysis of Gene Set Variation Analysis enrichment scores, transformed from TCGA, GEO, and CCLE transcriptomic profiles, revealed distinct immune subtypes. These subtypes were comprehensively characterized for their immune, mutational, and molecular features. Therapeutic sensitivity was assessed using computational prediction algorithms and validated via *in vitro* experiments. Finally, subtype-specific hub genes were identified through co-expression network analysis (WGCNA) and experimentally confirmed.

**Results:**

Three distinct immune subtypes were identified. Cluster II exhibited a survival advantage, high immune infiltration, and strong correlation with Basal-like tumors. In contrast, Cluster I aligned with Luminal subtypes and indicated poor prognosis. Therapeutic analysis revealed that Cluster II is most likely to benefit from immunotherapy. Additionally, five identified hub genes further confirmed the stability and accuracy of this immune stratification system.

**Conclusions:**

This study demonstrated that classifying identification of immune patterns for individual tumor patients could reveal the complexity of tumor immune microenvironment and further optimize precision immunotherapy.

## 1 Introduction

Breast cancer (BRCA) remains the leading cause of cancer-related mortality among women worldwide, distinguished by its profound clinical and biological heterogeneity (1–4). Recent cancer statistics in the United States indicate an estimated 290,560 new breast cancer cases and 43,780 deaths. Despite this high incidence, overall treatment outcomes are showing diminishing marginal improvements compared to earlier breakthroughs (3). While traditional paradigms have predominantly centered on the direct eradication of tumor cells, it is increasingly evident that sustainable therapeutic efficacy requires addressing the inherent complexity of the tumor microenvironment (TME), which often functions as a barrier to effective intervention. Crucially, the TME is characterized by a diverse infiltration of immune cells which can either drive or inhibit tumor growth complex regulatory mechanisms (5). This internal complexity, coupled with the biological heterogeneity of BRCA, explains why modern strategies—including targeted inhibitors and cancer immunotherapy (CIT)- frequently exhibit constrained efficacy and significant variability across molecular subtypes (6). Accordingly, establishing reliable immune targets and therapeutic strategies is imperative for advancing precision oncology (7). In this context, tumors are traditionally categorized into “hot” and “cold” tumors based on the abundance or lack of tumor-infiltrating lymphocytes (TILs) in TIME, and “hot” tumors are more sensitive to CIT (8). Accurate measurement of key markers related to immune response is the premise of CIT treatment, which strictly classifies patients and guides the selection of CIT therapy. To improve the efficacy of CIT, A further exploration of TIME is urgently needed, which may reveal more prognostic biomarkers and uncover novel targets for immunotherapy in BRCA.

Current oncological practice is increasingly dictated by molecular profiling, which underpins the shift toward highly stratified and personalized therapeutic interventions. With the completion of the Human Genome Project and the application of molecular biology techniques, breast cancer patients can be classified into four molecular subtypes, Lum A, Lum B, HER-2 and Basal, based on the level of detection of specific BRCA genes and protein levels (9). This typing reflects the biological behavior of BRCA more precisely and facilitates the selection and research of more tailored and personalized treatments. Basal patients are clinically more susceptible to benefit from immunotherapy (10). However, there are still many individuals who do not gain from immune checkpoint inhibitor therapy (ICB), thus necessitating a new typing method to predict the effect of ICB therapy.

**Figure 1:**
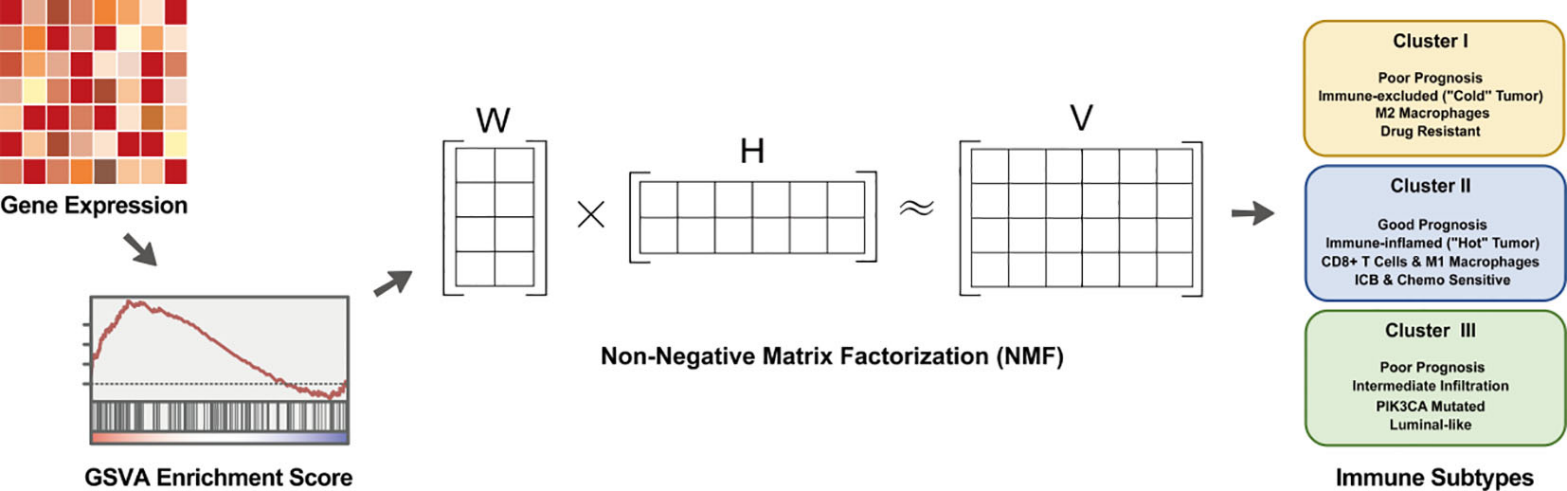
Schematic diagram of the workflow for identifying and characterizing breast cancer immune subtypes.

Herein, we constructed a comprehensive immunotyping model to decipher the heterogeneity of the tumor immune microenvironment (TIME) in breast cancer (**Figure 1**). In this study, we profiled the dynamic changes in immunological signature activities to identify three distinct immune subtypes, systematically mapping their TIME landscapes, mutational profiles, and correlation with intrinsic molecular subtypes. Crucially, moving beyond in silico analysis, we provided compelling experimental evidence to substantiate our therapeutic predictions. Through systematic screening and *in vitro* validation, we confirmed that Cluster II exhibits superior responsiveness to chemotherapeutic and targeted agents, distinguishing it as a prime candidate for immunotherapy. Finally, the identification of key hub genes via WGCNA further validated the stability and accuracy of this classification model, offering a reliable theoretical framework to facilitate personalized treatment and improve patient prognosis.

## 2 Materials and methods

### 2.1 Data collection and processing

We systematically collected breast cancer transcriptomic data and clinical annotations from multiple public repositories. The primary dataset, including mRNA expression profiles, somatic mutation data, and clinical information, was retrieved from The Cancer Genome Atlas (TCGA) (https://portal.gdc.cancer.gov/). Additionally, three independent breast cancer cohorts (GSE20685, GSE1456, and GSE20711) were obtained from the Gene Expression Omnibus (GEO) (http://www.ncbi.nlm.nih.gov/geo/). Transcriptomic data for 47 breast cancer cell lines were acquired from the Cancer Cell Line Encyclopedia (CCLE) (https://sites.broadinstitute.org/ccle/). Finally, a total of 4,872 immunologic gene sets were downloaded from the Molecular Signatures Database (MSigDB) (https://www.gsea-msigdb.org/gsea/msigdb/index.jsp). For the independent single-cell validation, the GSE176078 dataset was processed using a pipeline strictly consistent with the original study (11).

### 2.2 Gene set variation analysis and molecular subtyping

Gene Set Variation Analysis (GSVA) was employed to estimate the relative enrichment of specific gene sets across the sample population. Functional enrichment analyses, including Gene Ontology (GO) Molecular Function (MF) and Reactome pathways, were conducted using the ‘clusterProfiler’ R package (12, 13). For visualization and clustering of breast cancer samples, we utilized the ‘ComplexHeatmap’ and ‘CancerSubtypes’ packages (14). To identify distinct molecular patterns within the high-dimensional genomic data, we applied Non-negative Matrix Factorization (NMF). Specifically, Hartigan-Wong clustering and NMF-based consensus clustering were performed on the GSVA enrichment scores. For independent validation at the single-cell level, we introduced the GSE176078 dataset. Immune subtyping was conducted based on the bulk RNA-seq raw counts.

### 2.3 Estimation of immune infiltration in the tumor immune microenvironment

The ESTIMATE algorithm (applied via the ‘estimate’ R package) was utilized to calculate Immune and Stromal scores, thereby inferring tumor purity. To dissect the immune landscape further, the CIBERSORT algorithm was employed to quantify the relative proportions of 22 distinct tumor-infiltrating immune cell types using the LM22 signature matrix with standard parameters (15).

### 2.4 Analysis of somatic alterations

Tumor Mutation Burden (TMB) was calculated by quantifying the total number of non-synonymous mutations in breast cancer samples from the TCGA dataset. Subsequently, the differences in TMB among the three identified immune subtypes were compared. The ‘maftools’ R package was employed to process Mutation Annotation Format (MAF) files, and the tcgaCompare function was utilized to analyze the distinct mutation patterns and frequencies across subtypes (16).

### 2.5 Prediction of response to immunotherapy and chemotherapy

The expression levels of genes associated with Immune Checkpoint Blockade (ICB) were analyzed to infer potential clinical outcomes of immunotherapy (17). Specifically, the expression profiles of 10 well-established immune checkpoint genes were compared across the identified breast cancer immune subtypes. Furthermore, the Immunophenoscore (IPS)—which integrates four key immunogenic components: effector cells, immunosuppressive cells, MHC molecules, and immunomodulators—was utilized to predict patient response to immunotherapy (18). IPS data for breast cancer samples were retrieved from The Cancer Immunome Atlas (TCIA) (https://tcia.at/home), and differential analyses were conducted among the three subtypes. Chemosensitivity to common chemotherapeutic agents and targeted drugs was estimated using the ‘pRRophetic’ R package (19). The prediction was executed via the pRRopheticPredict function, based on ridge regression models constructed from cell line expression data.

### 2.6 Cell culture

Human breast cancer cell lines (HCC1937, MDA-MB-468, MDA-MB-231, T47D, MCF7, and BT549) were purchased from the Cell Bank of the Chinese Academy of Sciences (Shanghai, China). Cells were cultured in DMEM medium (HyClone, USA) supplemented with 10% Fetal Bovine Serum (FBS; PAA, Germany). Digestion and passaging were performed using Trypsin (Gibco, USA) and EDTA (Solarbio, Beijing, China). All cell lines were maintained in a CO_2_ incubator (Thermo Fisher Scientific, USA) at 37 °C in a humidified atmosphere containing 5% CO_2_.

### 2.7 Cell proliferation assay

Cell proliferation was assessed using the MTT assay kit (Solarbio, Beijing, China). Briefly, breast cancer cells were seeded into 96-well plates (5,000 cells per well) and allowed to adhere. Cells were treated with indicated drugs, while the control group received DMSO (Solarbio, Beijing, China). After 48 h of incubation, 20 μL of MTT solution was added to each well, and cells were incubated for 4 h. Subsequently, the supernatant was removed, and DMSO was added to dissolve the formazan crystals. The absorbance at 570 nm was measured using a microplate reader (BioTek, USA).

### 2.8 RNA extraction and quantitative real-time PCR

Total RNA was extracted from breast cancer cell lines using TRIzol reagent (Invitrogen, USA). Chloroform (Beijing Chemical Works, China), Isopropanol (Tianjin Fuyu Fine Chemical, China), and Ethanol (Tianjin Kemiou Chemical Reagent, China) were used for RNA isolation and purification. RNA was dissolved in DEPC-treated water (Sangon Biotech, Shanghai, China). Reverse transcription was performed to synthesize first-strand cDNA using a Reverse Transcription Kit (Vazyme Biotech, Nanjing, China) on a thermal cycler (Takara, Japan). Quantitative Real-Time PCR (qRT-PCR) was subsequently conducted using a SYBR Green qPCR Kit (Vazyme Biotech, Nanjing, China) on a Real-Time PCR System (Thermo Fisher Scientific, USA). The primers used were synthesized by Sangon Biotech (Shanghai, China).

### 2.9 Statistical analysis

Wilcoxon test was used for comparison between two groups of subtypes, one-way ANOVA and Kruskal-Wallis tests were used to conduct difference comparisons of three or more groups. Correlation of immunological subgroups with clinical characteristics or TMB was performed using the Chi-square test, and correlation coefficients were analyzed using Spearman’s correlation analysis. GraphPad Prism 7 software was used to perform unpaired Student’s t-tests to analyze differences between two groups. P < 0.05 was regarded as significant.

## 3 Results

### 3.1 Identification of breast cancer subtypes through immunologic gene set profiling

**Figure 2:**
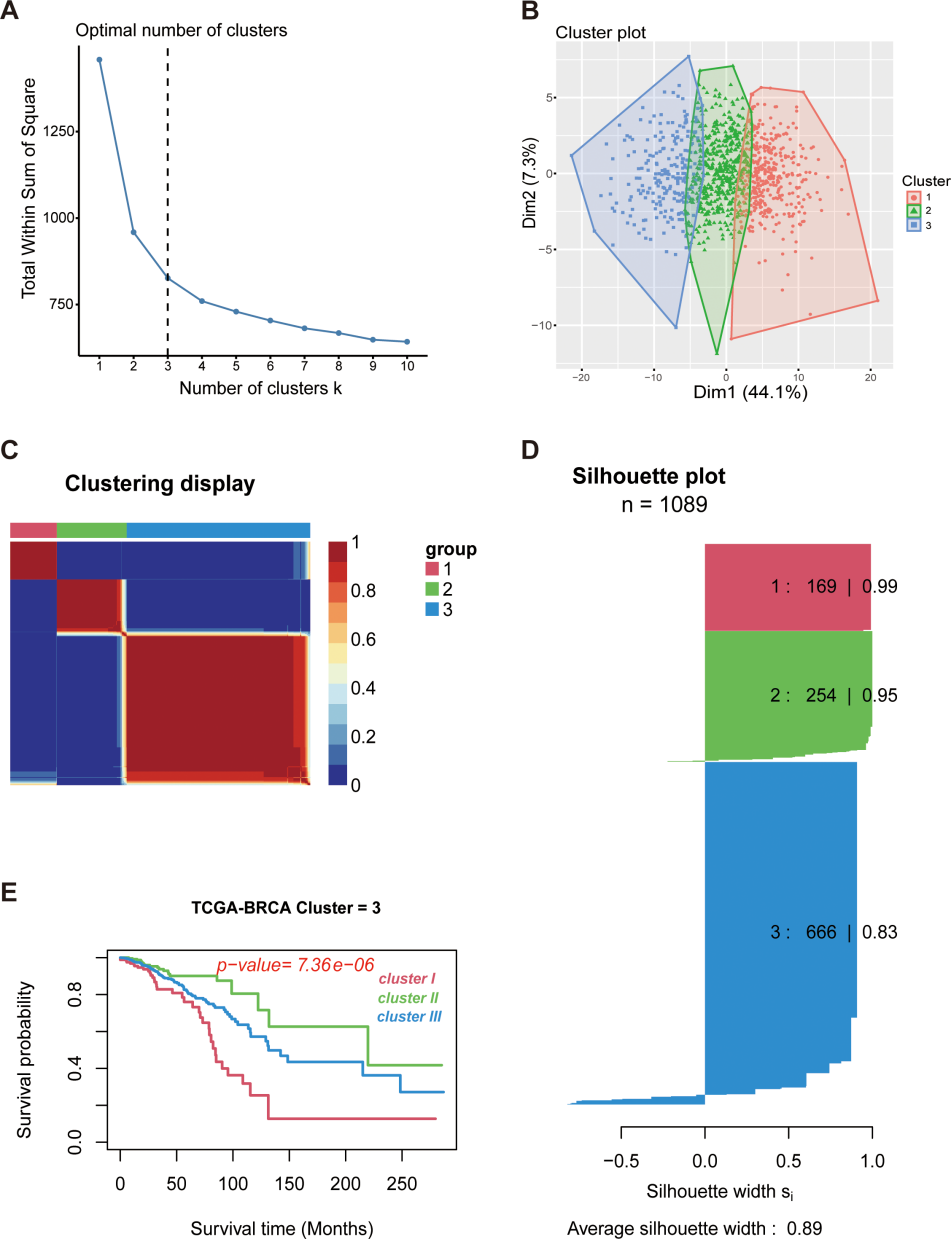
Identification of immune subtypes in breast cancer. **(A)** Selection of the optimal cluster number (k=3) based on the within-cluster sum of squares (WSS). **(B)** Cluster visualization of BRCA samples projected onto the first two dimensions. **(C)** NMF consensus clustering heatmap showing the stability of the three subtypes. **(D)** Silhouette width plot (n=1089) showing an average width of 0.89. **(E)** Kaplan-Meier curves showing distinct survival outcomes among the three subtypes (P = 7.36 × 10^-6^).

To delineate the immunologic landscape of breast cancer, we obtained 4,872 immunologic gene sets from ImmuneSigDB (20). Using these sets, Gene Set Variation Analysis (GSVA) was performed to calculate enrichment scores (ES) for the TCGA-BRCA cohort (n=1222) and external GEO validation datasets (n=576) (**Supplementary Tables S1**, **S2**). Unsupervised bi-clustering of the ES profiles revealed distinct immune patterns differentiating tumor tissues from normal breast samples (**Supplementary Figure S1**). Notably, significant heterogeneity was observed within the tumor samples, suggesting the potential for immune-based molecular subtyping. We determined the optimal number of clusters (k) by analyzing the total within-cluster sum of squares (WSS). As illustrated in **Figure 2A**, the cluster decision was guided by the point of maximum curvature at k=3, indicating that the gain in data representation yielded diminishing returns beyond this threshold. Subsequently, breast cancer samples were stratified into three distinct immune subtypes (Cluster I, II, and III) via Non-negative Matrix Factorization (NMF) consensus clustering (**Figures 2B, C**). The resulting stratification demonstrated high stability, evidenced by a silhouette width of 0.89 (**Figure 2D**), which underscores the robustness of the clustering in capturing intrinsic molecular heterogeneity. Survival analysis further highlighted the clinical relevance of these subtypes. Patients in Cluster II exhibited a significant survival advantage, whereas those in Clusters I and III were associated with markedly poorer prognoses (**Figure 2E**). To validate the robustness of this classification system, we applied the same stratification strategy to three independent external cohorts (GSE20685, GSE1456, and GSE20711). Consistent with the training set, the immune profiles and prognostic trends were preserved in the validation cohorts, with Cluster II consistently predicting favorable outcomes (**Supplementary Figure S2**). These results confirm the reproducibility and prognostic value of the identified immune subtypes.

### 3.2 Characterization of the tumor immune microenvironment across subtypes

**Figure 3:**
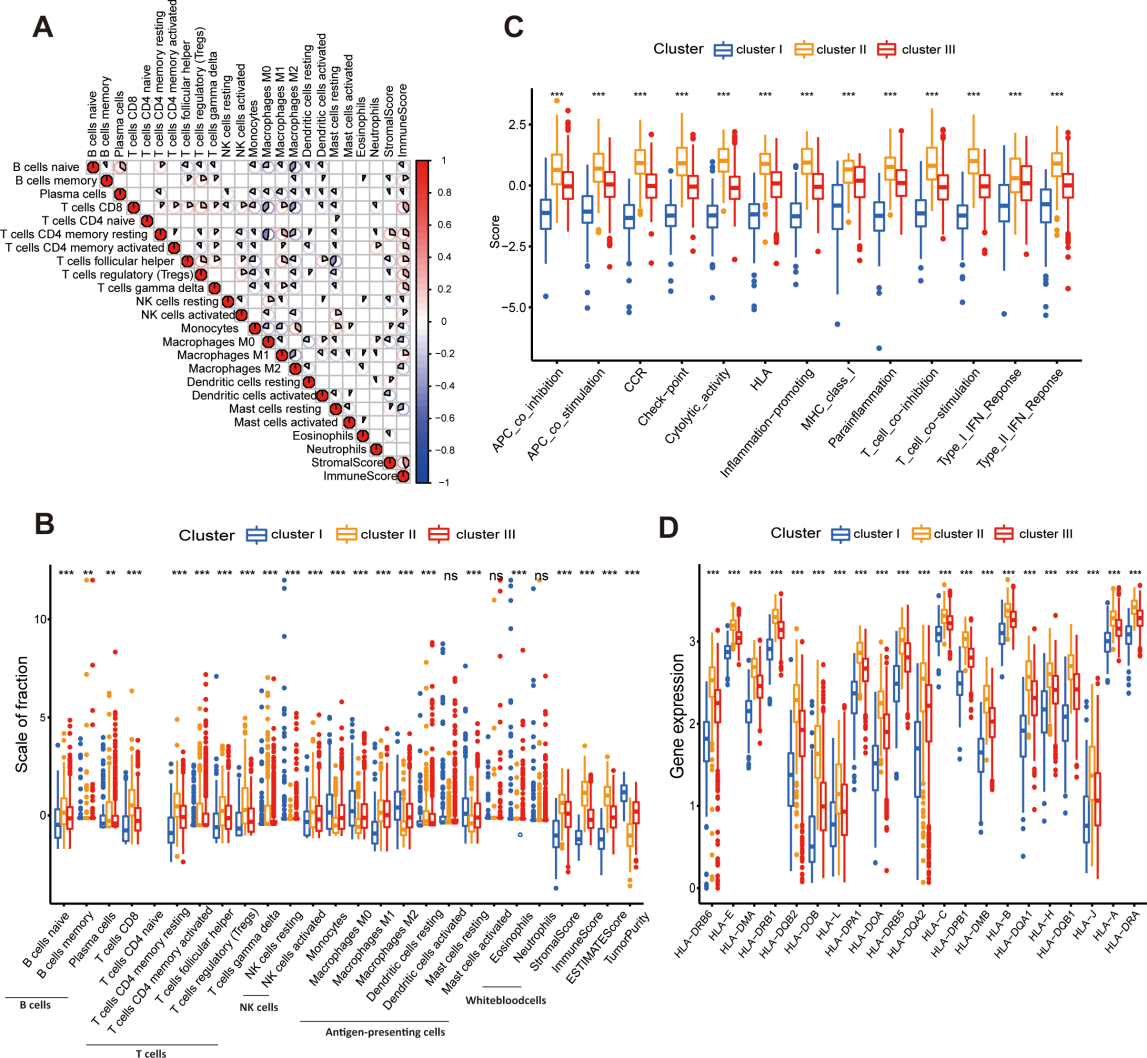
**(A)** Correlation analysis of infiltrating immune cells and immune/stromal scores. **(B)** Distribution of immune cell fractions, microenvironment scores (Immune/Stromal), and tumor purity across the three clusters. **(C)** Comparison of activity levels for 13 immune-related signatures/pathways. **(D)** Differential expression of HLA family genes among the subtypes. Statistical significance is indicated by asterisks (*P < 0.05; **P < 0.01; ***P < 0.001), and ns is P >= 0.05, indicating no significance).

To elucidate the immunological landscape of breast cancer, we quantified the relative abundance of 22 immune cell types and calculated immune scores using the CIBERSORT algorithm (**Figure 3A**). Correlation analysis revealed a complex interplay among immune cell populations: CD8+ T cells and activated CD4+ memory T cells exhibited strong positive correlations with overall immune scores. In contrast, these lymphocyte populations showed significant negative correlations with M0 and M2 macrophages, suggesting a mutual exclusivity between adaptive cytotoxic effectors and immunosuppressive myeloid cells within the TIME.

We next investigated the heterogeneity of immune infiltration across the three identified subtypes (**Figure 3B**; **Supplementary Figure S3A**). Significant variations in infiltration intensity were observed, with Cluster II displaying the highest overall immune infiltration, followed by Cluster III, whereas Cluster I exhibited the lowest levels. Specifically, Cluster II was characterized by an “immune-inflamed” phenotype, featuring significant enrichment of plasma cells, CD8+ T cells, activated CD4+ memory T cells, and pro-inflammatory M1 macrophages. Concurrently, this cluster showed depleted levels of immunosuppressive M0 and M2 macrophages. In sharp contrast, Cluster I displayed an “immune-excluded” phenotype, marked by a paucity of tumor-infiltrating lymphocytes (TILs) and a predominant enrichment of M2 macrophages.

To validate the immunogenicity of Cluster II, we examined the expression of antigen-presentation machinery and immune-related signaling. Consistent with its inflamed phenotype, Cluster II exhibited significantly enhanced activity across 13 key immune-related pathways (**Figure 3C**) and elevated expression of Human Leukocyte Antigen (HLA) genes (**Figure 3D**). These findings indicate that the superior survival outcomes observed in Cluster II are likely driven by robust antigen presentation and effective immune surveillance. Collectively, these distinct immune profiles-ranging from the M2-enriched/immune-excluded Cluster I to the lymphocyte-rich/immune-inflamed Cluster II—provide a biological rationale for the prognostic stratification and suggest potential differential responsiveness to immunotherapeutic interventions.

### 3.3 Molecular signatures and mutational landscapes of the immune subtypes

**Figure 4:**
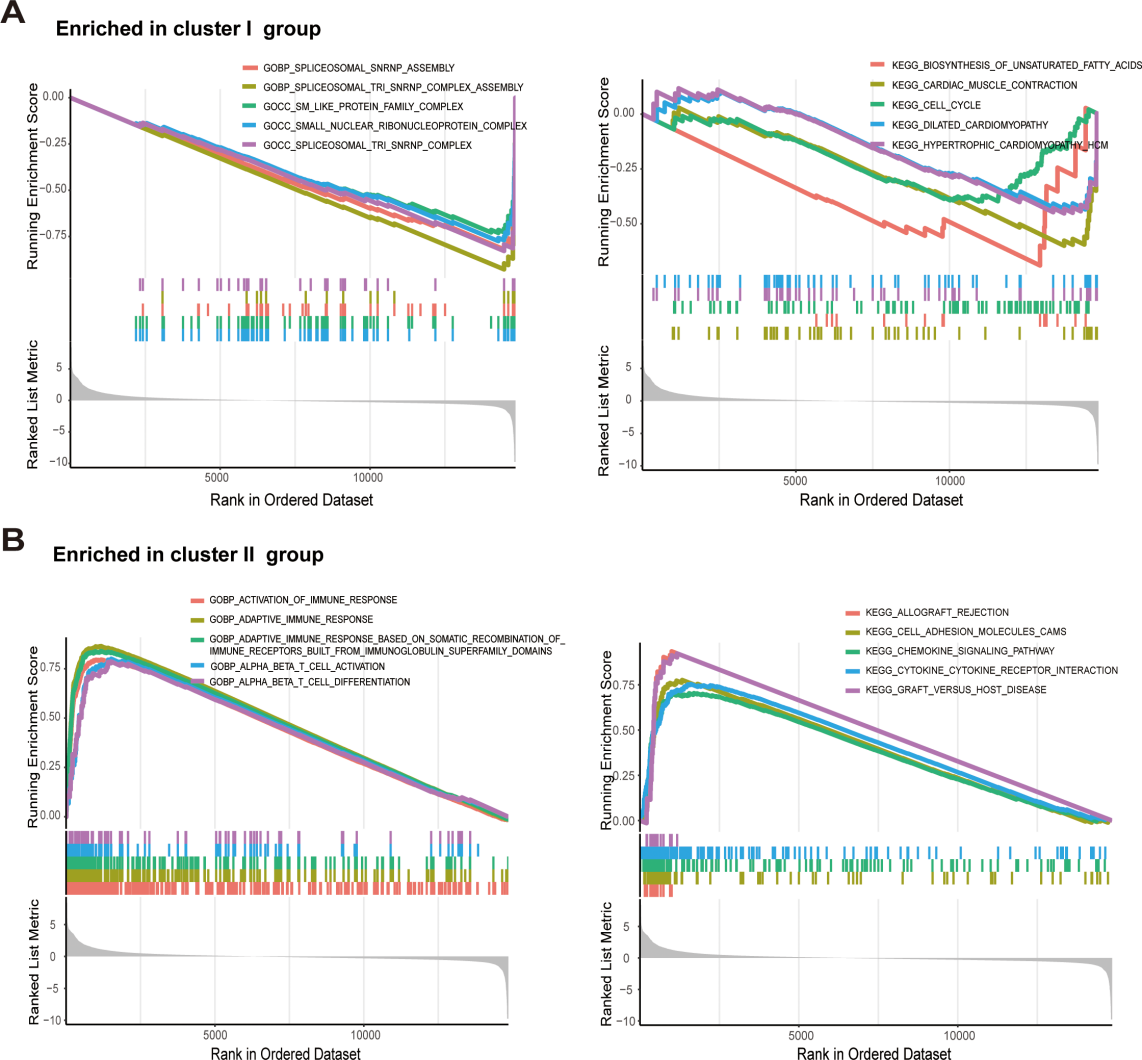
Uncovering potential biological processes associated with immune subtypes using GSEA. **(A)** Top GO terms and KEGG pathways enriched in Cluster I, showing an association with RNA processing and cell cycle progression. **(B)** Top GO terms and KEGG pathways enriched in Cluster II, demonstrating robust activation of immune-related signaling and adaptive immune responses.

To elucidate the biological mechanisms driving the distinct immune phenotypes, we performed Gene Set Enrichment Analysis (GSEA) based on Gene Ontology (GO) and KEGG pathways. This analysis revealed divergent functional programs between Cluster I and Cluster II. Cluster I was characterized by the significant enrichment of genes involved in fundamental cellular maintenance and proliferation, particularly RNA splicing and cell cycle progression (**Figure 4A**). This suggests that tumors within Cluster I are driven by aggressive proliferative capacity and hyperactive RNA processing machinery. In sharp contrast, Cluster II displayed a molecular profile predominantly defined by immunomodulation (**Figure 4B**).

**Figure 5:**
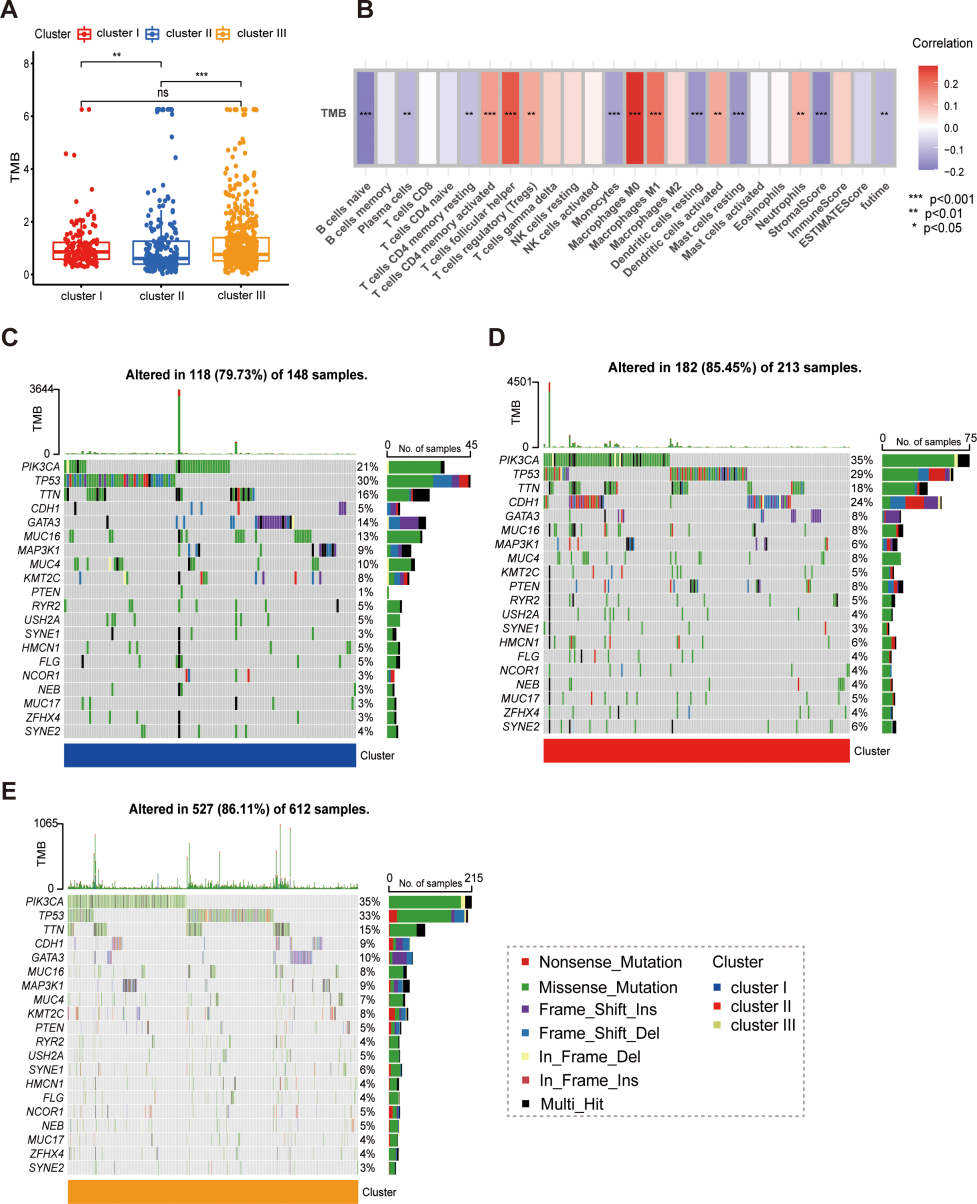
**(A)** Comparison of tumor mutational burden (TMB) across clusters; cluster II shows significantly lower TMB (**P < 0.01, ***P < 0.001). **(B)** Correlation heatmap of TMB with immune infiltration profiles and clinical prognosis. **(C–E)** Oncoplots displaying the somatic landscape of the top 20 driver genes for Cluster II **(C)**, Cluster I **(D)**, and Cluster III **(E)**. Mutation types are color-coded as indicated in the legend.

We identified a robust enrichment of pathways governing immune system activation, including adaptive immune responses and T-cell differentiation (specifically T cell activation). Furthermore, critical signaling cascades such as chemokine signaling and cytokine-cytokine receptor interactions were highly active in Cluster II. These transcriptomic signatures confirm that Cluster II represents an “immune-hot” phenotype characterized by intense immunological surveillance and signaling. We next interrogated the genomic instability of these subtypes by analyzing Tumor Mutational Burden (TMB). Interestingly, Cluster II exhibited a significantly lower TMB compared to the other subtypes (**Figure 5A**). Correlation analysis further revealed that TMB was associated with the infiltration levels of several immune cell subsets, including M0 macrophages and Regulatory T cells (Tregs) (**Figure 5B**). These observations align with findings by Andor et al. (21), suggesting that in this context, excessive genomic instability (high TMB) may correlate with a poorer prognosis. Finally, we characterized the somatic mutation landscape using waterfall plots of the top 20 driver genes (**Figures 5C–E**). Missense mutations were the predominant alteration type across all cohorts. PIK3CA and TP53 were the most frequently mutated genes universally. However, mutation frequencies varied by subtype: PIK3CA mutations were notably more prevalent in Clusters I and III (35%) compared to Cluster II (21%) (Note: Please check this comparison in your data). Distinctively, CDH1 mutations were substantially enriched in Cluster II (24%) relative to Clusters I and III (5% and 9%, respectively), a pattern similarly observed for PTEN. Conversely, Cluster I was distinguished by specific mutation patterns in the GATA3 and MUC16 genes. These distinct mutational profiles provide potential genomic biomarkers for tailoring targeted therapies and immunotherapy in breast cancer (22).

### 3.4 Association between immune subtypes and clinical characteristics

**Figure 6:**
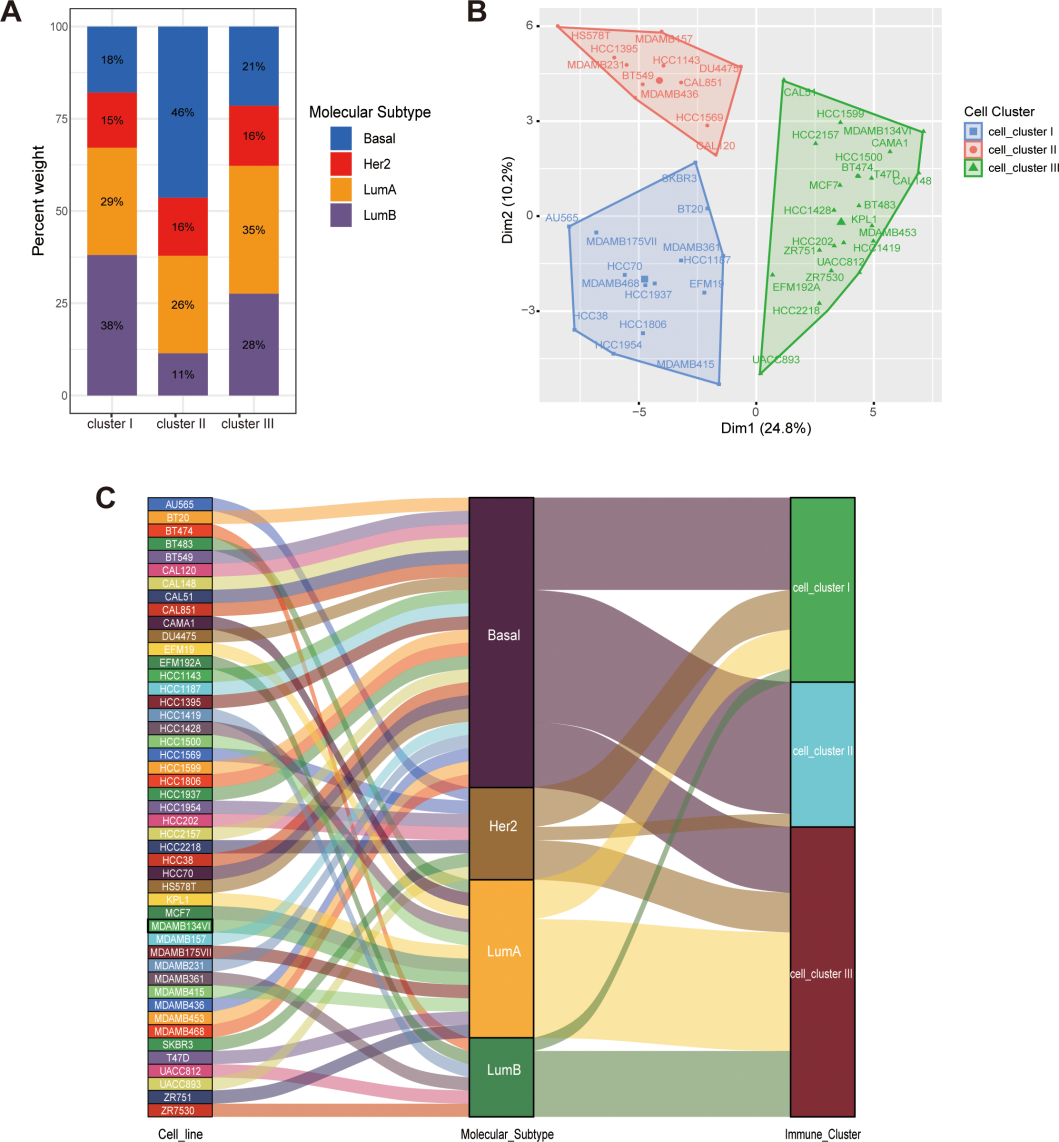
The relationship between immune subtypes and molecular subtypes. **(A)** Proportional distribution of PAM50 molecular subtypes within each immune cluster. **(B)** The immunological classification applied to breast cancer cell lines (CCLE cohort). **(C)** The mapping relationship between molecular subtypes and the identified cellular immune clusters.

To validate this association *in vitro*, we applied the same immunophenotyping strategy to 47 breast cancer cell lines from the CCLE database, classifying them into three corresponding cell clusters (**Figures 6A, B**) (23). An alluvial diagram visualized the concordance between immune classifications and molecular subtypes (**Figure 6C**). Notably, Cell-Cluster II consisted exclusively of HER2-enriched and Basal-like cell lines, with a marked predominance of the Basal-like subtype. Conversely, Luminal A and Luminal B cell lines were restricted to Cell-Clusters I and III. These findings demonstrate a high degree of concordance between immune profiles and intrinsic molecular subtypes at both the tissue and cellular levels. Specifically, Cluster II displays a distinct “Basal-like” immune signature, whereas Clusters I and III exhibit “Luminal-like” features.

### 3.5 Landscape of therapeutic sensitivity across distinct immune subtypes

**Figure 7:**
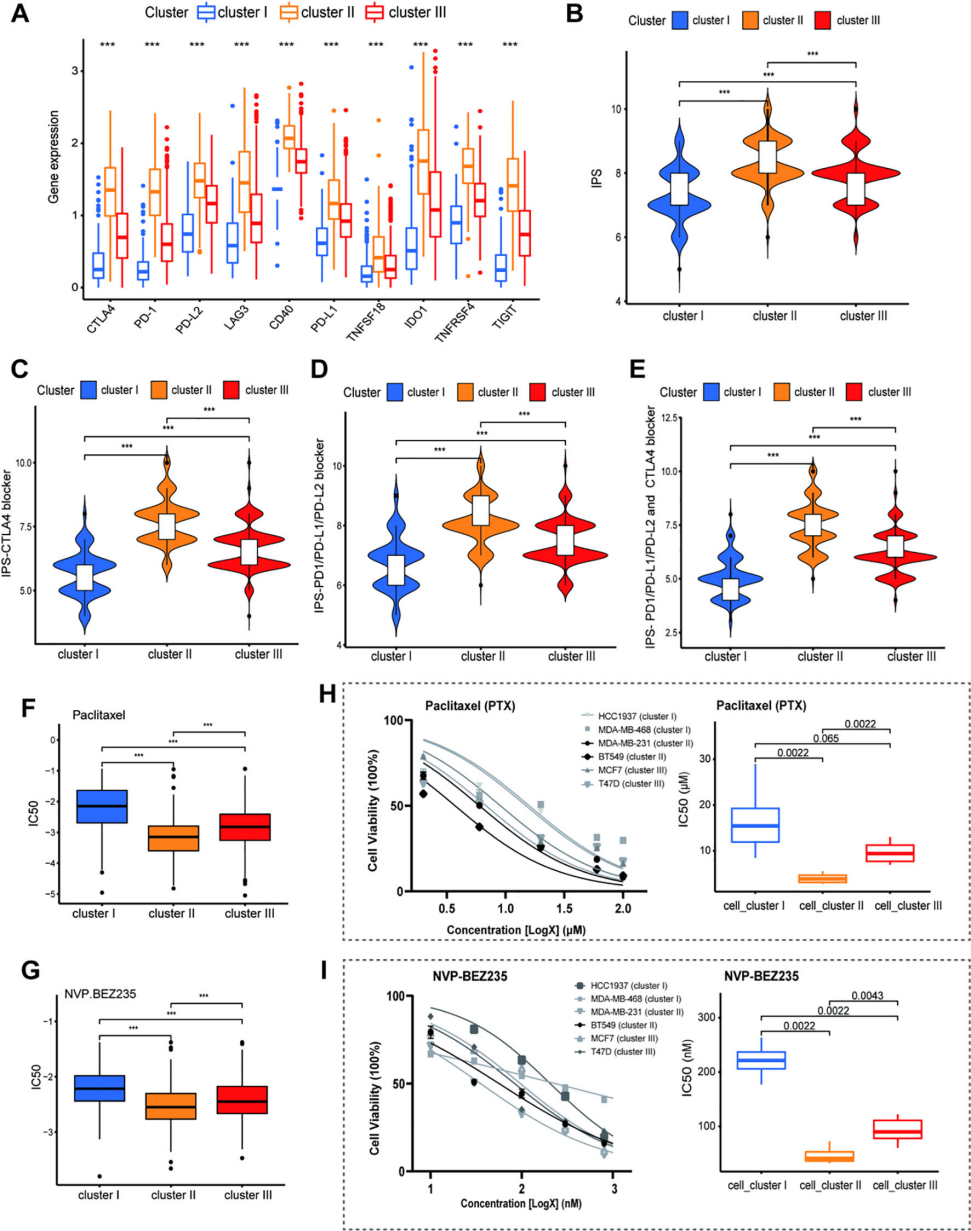
**(A)** Expression levels of 10 immune checkpoint genes. **(B–E)** Violin plots showing IPS scores for different ICB treatment scenarios. **(F, G)** Predicted IC50 values for Paclitaxel and NVP-BEZ235; Cluster II shows lower values (higher sensitivity). **(H)** Dose-response curves (left) and IC_50_ box plots (right) for Paclitaxel (PTX) treatment across 3 breast cancer cell clusters (cluster I: HCC1937, MDA-MB-468; cluster II: MDA-MB-231, BT549; cluster III: MCF7, T47D). **(I)** Dose-response curves (left) and IC_50_ box plots (right) for NVP-BEZ235 treatment across the same 3 cell clusters. Statistical significance is indicated by asterisks (***P < 0.001).

Our immune subtyping framework may offer novel insights for optimizing clinical treatment strategies in breast cancer. To evaluate the potential response to immunotherapy, we first analyzed the expression profiles of 10 key immune checkpoint-related genes (including CTLA4, CD274, PDCD1, LAG3, CD40, and TIGIT). Notably, all examined checkpoint genes were significantly upregulated in Cluster II (**Figure 7A**), suggesting that patients in this subgroup may exhibit enhanced sensitivity to Immune Checkpoint Blockade (ICB) therapy.

To further quantify this potential, we utilized the Immunophenoscore (IPS) algorithm as a predictive surrogate for ICB response. Consistent with the gene expression data, Cluster II samples exhibited significantly higher scores across all IPS categories—including IPS-CTLA4, IPS-PD1/PD-L1/PD-L2, and dual blockade scores (**Figures 7B–E**). These findings reinforce the hypothesis that patients classified into Cluster II are the most suitable candidates for immunotherapy. We next investigated the association between immune subtyping and chemotherapeutic sensitivity by estimating IC50 values. For conventional chemotherapeutic agents—including Paclitaxel (PTX) (**Figure 7F**), Gemcitabine, Doxorubicin, and Vinorelbine (**Supplementary Figure S3B**)—Cluster II demonstrated substantially lower IC50 values compared to other clusters, indicating higher drug sensitivity. Similarly, analysis of targeted therapeutics revealed distinct response patterns. Cluster II was associated with significantly enhanced sensitivity (lower IC50) to multiple inhibitor classes, including PARP inhibitors (AZD-2281, ABT-888) (**Supplementary Figure S3C**), HSP90 inhibitors (AUY-922) (**Supplementary Figure S3D**), VEGFR/CDK inhibitors (GCP-60474) (**Supplementary Figure S3E**), as well as PI3K/mTOR pathway inhibitors (Rapamycin, GDC-0941, NVP-BEZ235, and JW-7-25-1) (**Figure 7G**, S3F-H).

Finally, *in vitro* validation using MTT assays confirmed these computational predictions. Cell lines corresponding to Cluster II exhibited significantly higher sensitivity to PTX (**Figure 7H**; **Supplementary Figure S4A**) and the dual PI3K/mTOR inhibitor NVP-BEZ235 (**Figure 7I**; **Supplementary Figure S4B**) compared to cell lines classified as Clusters I and III.

### 3.6 Characterization of subtype-specific hub genes across immune subtypes

**Figure 8:**
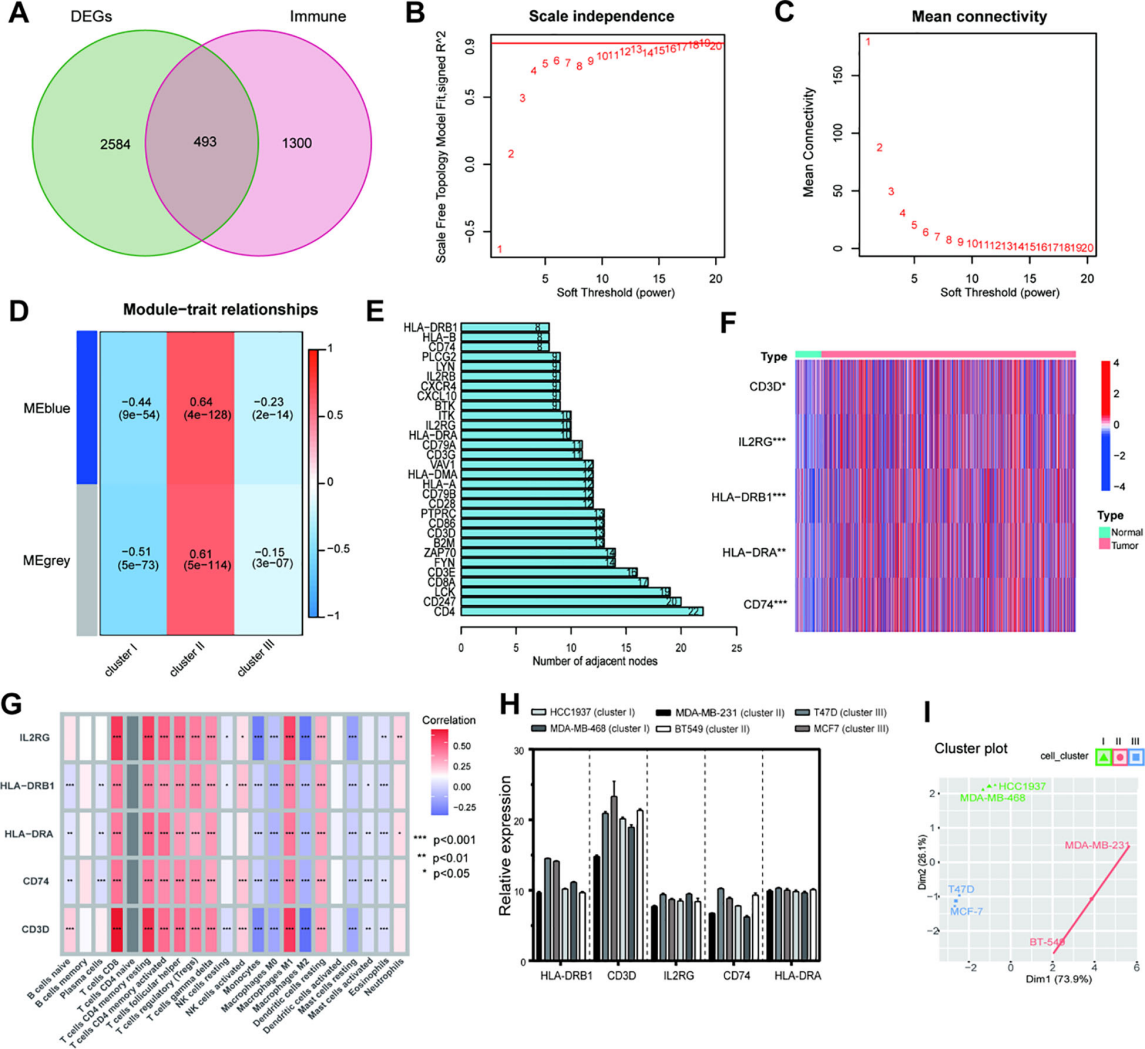
Screening and validation of key immune hub genes. **(A)** Selection of 493 immune-related DEGs for WGCNA. **(B, C)** Analysis of network topology to select the optimal soft-thresholding power (beta=13). **(D)** Heatmap of the correlation between gene modules and immune subtypes; the blue module is specific to Cluster II. **(E)** The top 30 hub gene candidates in the blue module identified by connectivity. **(F)** Expression differences of the five validated hub genes in tumor versus normal tissues. **(G)** Correlation analysis between hub genes and tumor-infiltrating immune cells. **(H)** qRT-PCR validation of hub gene expression across six breast cancer cell lines. **(I)** Classification of cell lines into immune clusters using hub gene enrichment scores. (*P < 0.05, **P < 0.01, ***P < 0.001).

**Figure 9:**
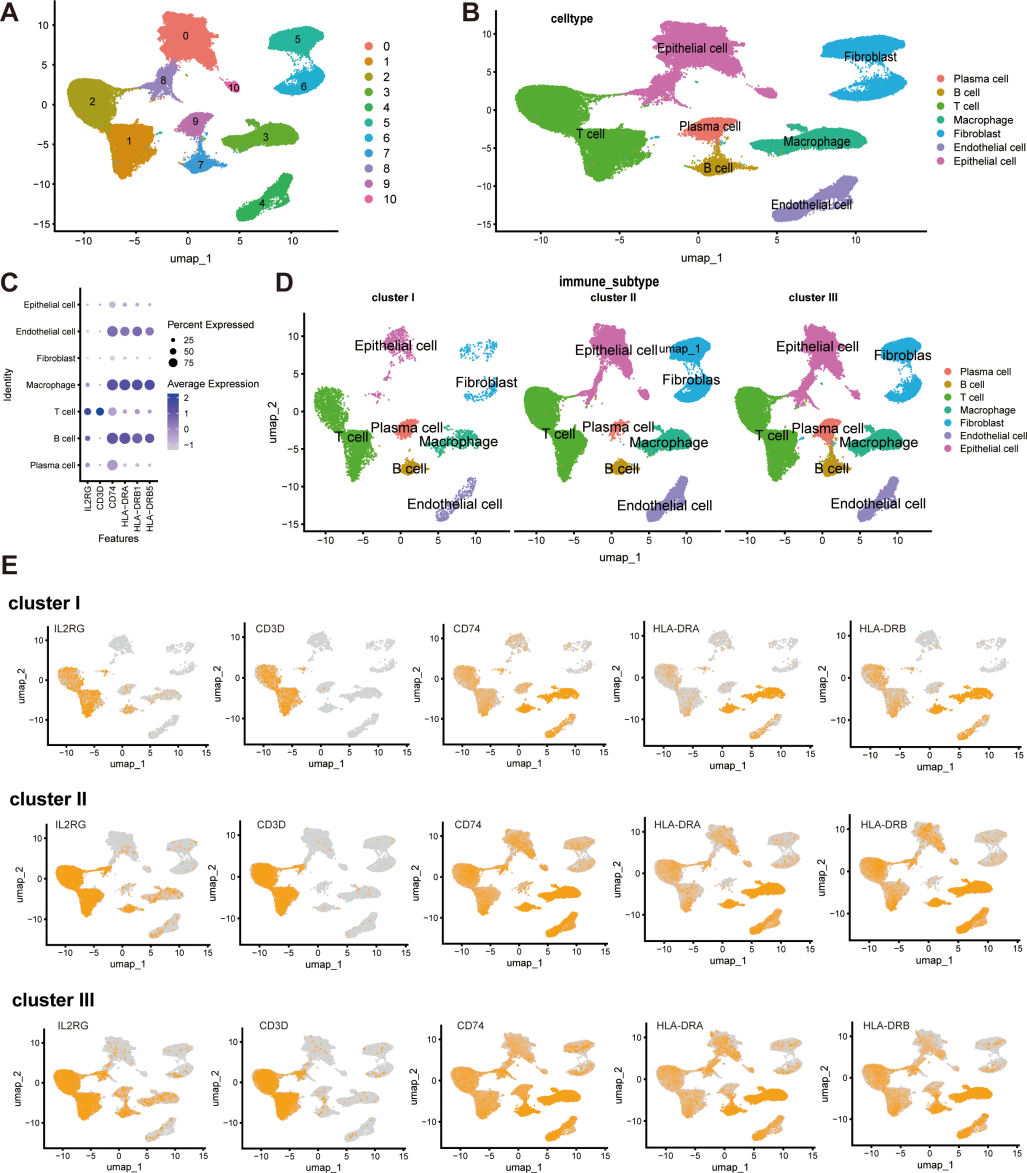
Single-cell RNA sequencing validation of hub gene expression patterns across immune subtypes. **(A)** UMAP plot illustrating 11 unsupervised cell clusters identified from the breast cancer single-cell dataset. **(B)** UMAP visualization color-coded by annotated cell types, including T cells, B cells, macrophages, epithelial cells, fibroblasts, and endothelial cells. **(C)** Dot plot displaying the expression levels of the five hub genes IL2RG, CD3D, CD74, HLA-DRA, HLA-DRB5 across different cell types. **(D)** Cell type distribution across Cluster I, Cluster II, and Cluster III. **(E)** Hub gene expression in each immune subtype.

To identify key drivers underlying the superior immune activity of Cluster II, we screened 493 immune-related genes from 3,077 DEGs for WGCNA (**Supplementary Figures S3I**, **S8A**) (24). A scale-free co-expression network was constructed (beta=13), identifying a blue module significantly correlated with Cluster II (**Figures 8B–D**; **Supplementary Figure S3J**). After protein-protein interaction (PPI) analysis and prognostic filtering (**Supplementary Figures S3K, L**; **S8E**), five pivotal hub genes—IL2RG, HLA-DRB, HLA-DRA, CD74, and CD3D—were identified (**Figure 8F**). To further elucidate the biological roles of these hub genes, we integrated single-cell RNA sequencing data (GSE176078). We applied our NMF-based classification framework to the bulk-level expression profiles of each patient within this dataset (**Figures 9A, B**). Following this patient-level stratification, we performed high-resolution analysis to evaluate the expression patterns of our hub genes at the single-cell level. Our analysis confirmed that IL2RG and CD3D are specifically and highly expressed in T-cell populations, rather than in epithelial or endothelial cells (**Figure 9C**). In contrast, MHC-II related molecules HLA-DRA and CD74—essential for antigen presentation to CD4+ T cells—showed significantly lower expression in Cluster I compared to Clusters II and III. This suggests that the low expression of these molecules in Cluster I mediates a state of “immune escape,” preventing effective T-cell recruitment and activation. (**Figures 9D, E**). This biological gradient suggests that the low expression of these molecules in Cluster I mediates an immune-cold phenotype, characterized by defective antigen presentation and failed T-cell recruitment. Thus, this immunosuppressive environment potentially contributes to the unfavorable prognosis observed in Cluster I. Correspondingly, bulk-tissue correlation analysis confirmed that these hub genes robustly associate with anti-tumor effectors (CD8+ T cells) while negatively correlating with immunosuppressive M2 macrophages (**Figure 8G**). Beyond characterizing their biological origins, we evaluated the transferability of these hub genes *in vitro*. Although these genes are immune-derived, their expression patterns across six breast cancer cell lines correctly stratified them into their respective clusters: Cluster I (HCC1937/MDA-MB-468), Cluster II (BT549/MDA-MB-231), and Cluster III (MCF7/T47D) (**Figures 8H, I**). The high concordance between our cell-line-based classification and the original patient-derived model provides compelling evidence for the accuracy and translational potential of our immunophenotyping framework.

## 4 Discussion

Breast cancer continues to represent a paramount global public health challenge, persisting as the leading cause of cancer-related morbidity and mortality among women. Due to the high heterogeneity of tumor microenvironmental phenotypes and tumor cell marker genotypes, clinical features, malignancy, and prognosis vary significantly among molecular subtypes, and the corresponding clinical outcomes are often unsatisfactory. Molecular subtypes of breast cancer are strongly associated with prognosis and treatment regimens. Currently, a comprehensive treatment strategy is commonly adopted in the clinic, including major modalities such as surgery, radiotherapy, chemotherapy, and endocrine therapy. In recent years, the rise of immunotherapy has ushered in a new era of oncology treatment, showing excellent prospects in improving the prognosis of a wide range of tumors. Numerous studies have demonstrated the effectiveness of immunotherapy in patients with advanced breast cancer (25, 26). However, a substantial proportion of patients still fail to derive benefit from these interventions. As such, the identification of effective biomarkers is crucial to facilitate the development of immunotherapy for breast cancer.

Increasing studies have suggested that the regulation of immune infiltrating cells acts as a decisive driver of tumor progression and anti-tumor immunity (27, 28). However, numerous researches have focused only on a single TIME subpopulation or a few immune-related genes, and the comprehensive landscape of TIME mediated by subtypes with distinct immune profiles has not yet been fully elucidated in breast cancer. In this research, we presented a comprehensive picture of the changes in the activity of immunological signature gene sets in breast cancer and identified three immune subtypes with distinct immune activities (**Table 1**). These immunological subtypes possessed distinct characteristics corresponding to different clinical therapeutic effects (**Table 2**). Cluster II was the most immunologically active of the three types and exhibited higher tumor lymphocyte infiltration, thus corresponding to a better prognosis and improved immunotherapeutic efficacy. In contrast, Cluster I, characterized by higher infiltration of M2 macrophages, was associated with a poor prognosis. Tumor-associated macrophages (TAMs) are a major component of the TIME in breast cancer. Macrophages exhibit a high degree of plasticity in response to various external signals, engaging in both innate and adaptive immune responses to regulate numerous factors within the TIME (29). The two main macrophage populations, M1 and M2, perform divergent functions and are differentiated/polarized by distinct signaling pathways. M2-polarized macrophages exert pro-angiogenic and immunosuppressive functions, whereas M1 macrophages perform anti-tumor functions. The inherent plasticity of macrophages implies that M2 macrophages can be stimulated to alter their phenotype, serving as a potential therapeutic strategy. Reprogramming M2 macrophages with various pharmacological agents can restore pro-immune and anti-tumor activities. Therefore, macrophage-targeted therapy represents a promising novel strategy for breast cancer treatment (30–32). Additionally, Zhang et al. identified two different types of M2-like TAMs and developed a prognostic signature revealing their diversity in breast cancer, highlighting their correlation with immune status and prognosis, which offers novel concepts for tailoring breast cancer treatment (33).

**Table 1:** Summary of the distinct characteristics of the three immune subtypes.

Cluster I	Cluster II	Cluster III
16%	23%	61%
Poor prognosis	Good prognosis	Average prognosis
Less TILs and more immunosuppressive cells infiltration (immune-excluded phenotype)	TILs infiltration and activation (immune-inflamed phenotype)	Moderate immune cells infiltration
High TMB, GATA3 and MUC16 mutations	Low TMB, PIK3CA, CDH1 and PTEN mutations	Middle TMB, PIK3CA and TP53 mutations
Lum-like	Basal-like	Lum-like
	Better suited for immunotherapy and Chemotherapy	

**Table 2:** Clinical characteristics of the three immune subtypes in breast cancer.

Parameter		Immune subtype (n, %)			P value
		Cluster I	Cluster II	Cluster III	
Age	<=65	95(68.35)	166(85.13)	404(70.38)	**0.0001**
	>65	44(31.65)	29(14.87)	170(29.62)	
Gender	FEMALE	136(97.84)	194(99.49)	567(98.78)	0.3992
	MALE	3(2.16)	1(0.51)	7(1.22)	
Stage	Stage I	16(11.51)	43(22.05)	100(17.42)	0.1064
	Stage II	93(66.91)	105(53.85)	334(58.19)	
	Stage III	29(20.86)	45(23.08)	126(21.95)	
	Stage IV	1(0.72)	2(1.03)	14(2.44)	
T	T1	27(19.42)	64(32.82)	144(25.09)	**0.0239**
	T2	93(66.91)	101(51.79)	345(60.1)	
	T3	12(8.63)	27(13.85)	63(10.98)	
	T4	7(5.04)	3(1.54)	22(3.83)	
M	M0	138(99.28)	193(98.97)	560(97.56)	0.2503
	M1	1(0.72)	2(1.03)	14(2.44)	
N	N0	66(47.48)	99(50.77)	284(49.48)	0.55
	N1	52(37.41)	64(32.82)	185(32.23)	
	N2	17(12.23)	18(9.23)	68(11.85)	
	N3	4(2.88)	14(7.18)	37(6.45)	

Bold values indicate statistical significance (P < 0.05).

Different immune subtypes exhibit distinct molecular characteristics. In GSEA, we found that Cluster II was enriched for numerous immune-related pathways—including activation of immune response, T-cell activation and differentiation, and chemokine signaling pathways—indicating the strongest immunoreactivity and the best prognosis. This suggests that the state of immune activity plays a critical role in opposing tumor progression and positively influences the clinical outcome of immune-based strategies. To further understand the immune profile of these subtypes, we investigated their mutational landscapes. We found a negative correlation between Tumor Mutational Burden (TMB) and patient prognosis in the TCGA-BRCA cohort. Moreover, Cluster II exhibited the highest CDH1 mutation rate. E-cadherin protein (CDH1 gene) integrity is fundamental to epithelial polarization and differentiation. Deregulation of E-cadherin function plays a crucial role in breast cancer metastasis and might provide new perspectives for targeted cancer therapy and immunotherapy (34).

The evolution of precision oncology emphasizes that traditional intrinsic subtypes, while fundamental, may not fully capture the complex immune landscape necessary for personalized treatment (35). In alignment with this trend, while drawing inspiration from broader immune subtyping frameworks (36, 37), our study seeks to adapt and refine these immunological concepts specifically within the breast cancer landscape. By integrating an immune-signature dimension, our subtyping strategy enriches the conventional PAM50-based classification. In terms of sample-level and cellular classification, Cluster II consisted mainly of the Basal-like type, whereas Clusters I and III exhibited “Luminal-like” characteristics. However, our single-cell analysis reveals that Cluster I is uniquely defined by a more profound functional impairment of the immune microenvironment, specifically through the deficit in antigen-presentation molecules (HLA-DRA/CD74). Clinically, patients with Basal-type breast cancer, which is more heavily infiltrated by tumor lymphocytes than other molecular subtypes, are considered potential candidates for immunotherapy. Triple-negative breast cancer (TNBC) is currently the most suitable subtype for immunotherapeutic strategies due to high levels of tumor-infiltrating lymphocytes (TILs) and PD-L1 expression compared to other subtypes (38). It was further found that combining molecular subtypes with their unique immune characteristics leads to new avenues for therapeutic development. The recent approval of sacituzumab govitecan-hziy, an antibody-drug conjugate (ADC), for the treatment of advanced TNBC illustrates the therapeutic advances that may result from these molecular discoveries (39).

Furthermore, the key genes with high immunoreactivity identified based on the immunophenotyping model (IL2RG, HLA-DRB, HLA-DRA, CD74, and CD3D) also corresponded to distinct prognostic and immunological profiles. These genes serve as potential markers for prognosis and immunotherapeutic efficacy in breast cancer. Elevated expression of IL2RG (also known as CD132) has been associated with improved breast cancer prognosis and increased immune cell infiltration (40). Similarly, the HLA system plays a key role in the escape of tumor cells from immune surveillance; HLA-DRB and HLA-DRA may provide new targets for immunotherapy. Additionally, CD74 expression was strongly correlated with PD-L1 expression, suggesting that CD74 may have predictive value for immune checkpoint therapy. Another study also showed that CD3D upregulation may increase T-cell infiltration in the TIME and induce anti-tumor immunity by activating T lymphocytes, with its expression showing a strong positive correlation with immune checkpoints (41).

In the era of precision medicine, stratified management and individualized treatment based on different molecular subtypes will certainly become the mainstream paradigm. There is a growing demand to use molecular signatures of individual patients to assess the potential applicability of therapeutic interventions. The advancement and widespread application of cancer genomics have had a significant impact on our understanding of the molecular heterogeneity between different subtypes of the same tumor, advancing cancer diagnosis, treatment, and drug development. However, our study focused mainly on the transcriptomic and genomic levels, which is far from sufficient for comprehensive precision treatment. Within the vision of precision cancer therapy, cancer proteogenomics brings new perspectives to assist in identifying potentially actionable signatures that more easily define tumor subtypes for predicting therapeutic effects, ultimately leading to the development of new protocols for refractory cancers (42).

## 5 Conclusions

By leveraging multi-omics data from TCGA, GEO, and CCLE, we constructed a stable immunophenotyping model for breast cancer. This classification revealed significant heterogeneity in the tumor immune microenvironment, with Cluster II emerging as a highly active ‘immune-inflamed’ phenotype. Patients within this cluster are predicted to derive the greatest benefit from immunotherapy and combined therapeutic regimens. Consequently, this subtype model serves as a valuable prognostic tool, offering a new perspective for refining precision oncology and improving clinical management strategies.

## Data Availability

Publicly available datasets were analyzed in this study. This data can be found here: TCGA (https://portal.gdc.cancer.gov/) and NCBI Gene Expression Omnibus (GEO) under accession codes GSE20685, GSE1456, GSE20711, and GSE176078.
