# Supplementary material for: Integrated characterization of distinct immune subtypes linked to drug responsiveness in breast cancer
Source: Front Immunol. 2026 May 5;17:1795917. doi: 10.3389/fimmu.2026.1795917 (PMC13183846; doi:10.3389/fimmu.2026.1795917)
Supplement: Supplementary file 1 [file SupplementaryFile1.docx]

**Supporting Information**

**Integrated Characterization of Distinct Immune Subtypes Linked to Drug Responsiveness in Breast Cancer**

Min Liu^1, 2^, Meina Yang^2,3^ and Bo-shi Fu^2, 3,^ *

1 Department of Pharmacy, the First Affiliated Hospital of Xi’an Jiaotong University, Xi’an, China;

2 Department of Pharmacology, School of Pharmacy, China Medical University, Shenyang, China;

3 Liaoning Key Laboratory of Molecular Targeted Anti-Tumor Drug Development and Evaluation, Liaoning Cancer Immune Peptide Drug Engineering Technology Research Center, Shenyang, China;

***** Correspondence: fuboshi@whu.edu.cn;

**Materials Methods and Instrumentation.** The following solvents, compounds, and reagents were all commercially available:

Cell Lines: The human breast cancer cell lines HCC1937, MDA-MB-468, MDA-MB-231, T47D, MCF-7, and BT-549 were obtained from the Cell Bank of the Chinese Academy of Sciences (Shanghai, China).

Cell Culture Reagents: DMEM medium was from HyClone (Logan, UT, USA). Fetal Bovine Serum (FBS) was from PAA Laboratories (Cölbe, Germany). Trypsin (0.25%) was from Gibco (Thermo Fisher Scientific, Waltham, MA, USA). EDTA and Dimethyl sulfoxide (DMSO) were from Solarbio Science & Technology Co., Ltd. (Beijing, China).

Molecular Biology Reagents & Kits: TRIzol reagent was from Invitrogen (Thermo Fisher Scientific, USA). DEPC-treated water and qPCR primers were from Sangon Biotech Co., Ltd. (Shanghai, China). The Reverse Transcription Kit and SYBR Green qPCR Kit were from Vazyme Biotech Co., Ltd. (Nanjing, China).

Chemical Reagents: Chloroform was from Beijing Chemical Works (Beijing, China). Disodium hydrogen phosphate (Na₂HPO₄) and potassium chloride (KCl) were from Tianjin Ruijin Regent Chemicals Co., Ltd. (Tianjin, China). Potassium dihydrogen phosphate (KH₂PO₄) was from the same supplier (Tianjin Ruijin Regent Chemicals). Isopropanol was from Tianjin Fuyu Fine Chemical Co., Ltd. (Tianjin, China). Absolute ethanol was from Tianjin Kemiou Chemical Reagent Co., Ltd. (Tianjin, China). Sodium chloride (NaCl) was from Tianjin Hengxing Chemical Reagent Manufacturing Co., Ltd. (Tianjin, China).

Assay Kits and Inhibitors: The MTT assay kit was from Solarbio Science & Technology Co., Ltd. (Beijing, China). The chemical inhibitors Paclitaxel and NVP-BEZ235 were purchased from TargetMol Chemicals Inc. (Boston, MA, USA).

Major Instruments: A class II biological safety cabinet and a CO₂ incubator were from Thermo Fisher Scientific (Waltham, MA, USA). A 37°C constant temperature shaker was from a domestic supplier (China). A bacterial incubator was from Thermo Fisher Scientific (USA). An inverted microscope and imaging system were from OLYMPUS Corporation (Tokyo, Japan). A microplate reader was from BioTek Instruments, Inc. (Winooski, VT, USA). A thermal cycler for PCR and reverse transcription was from Takara Bio Inc. (Kusatsu, Japan). A real-time quantitative PCR system was from Thermo Fisher Scientific (USA).

**Table S1** **Enrichment scores of immune-related gene sets in the TCGA-BRCA cohort (n=1222).**

**Table S2 Validation of immune signature enrichment scores in the external GEO cohort (n=576).**

**
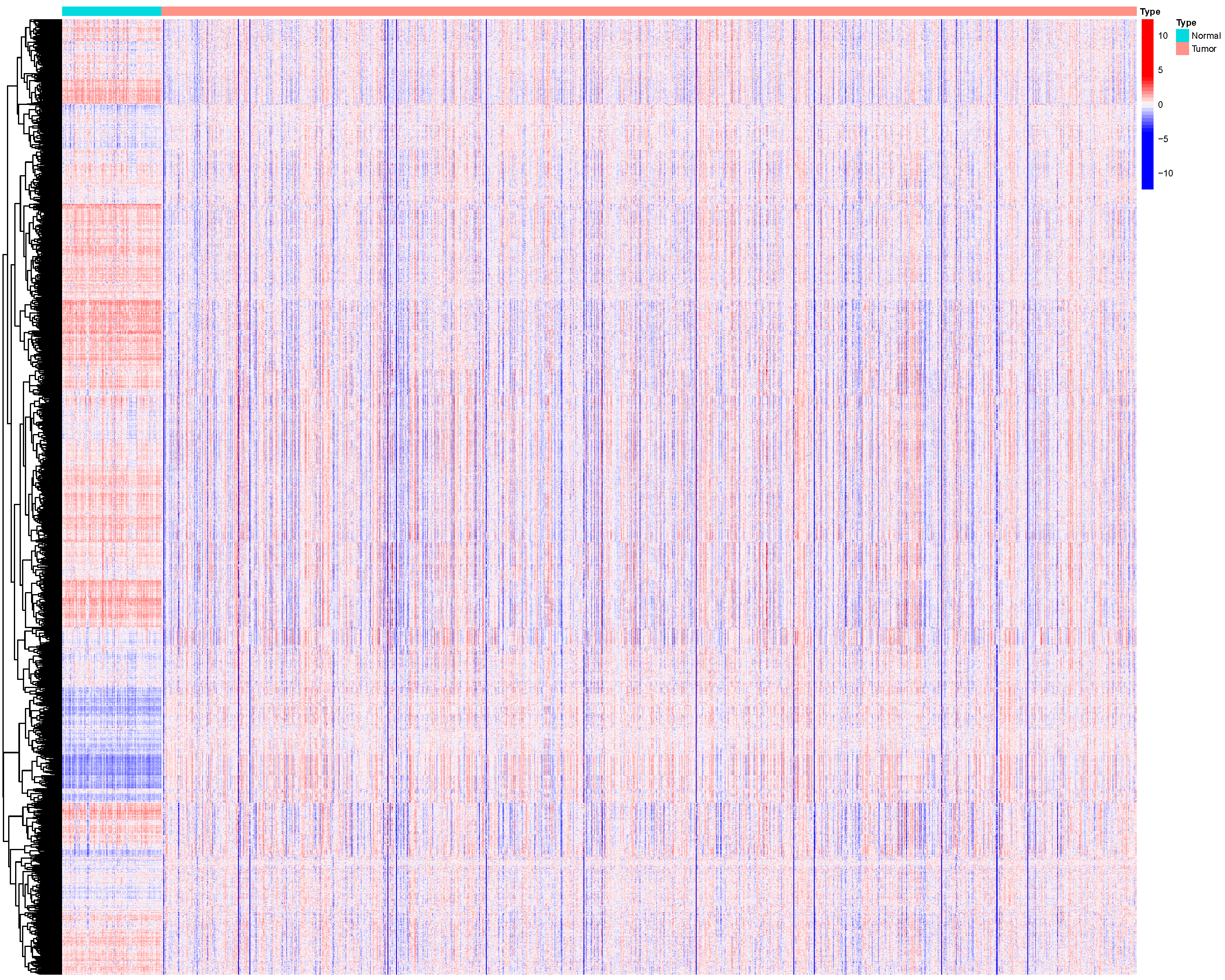
**

**Figure S1.  Distinct immune gene set enrichment profiles distinguish breast tumor tissues from adjacent non-tumor samples.** Heatmap depicting the Gene Set Variation Analysis (GSVA) enrichment scores of immune-related gene sets across samples in the TCGA-BRCA cohort (n=1,222). Rows represent GSVA enrichment score (ES), and columns represent individual samples. Samples are grouped and color-coded at the top as tumor (red) or normal (blue) tissues.

**
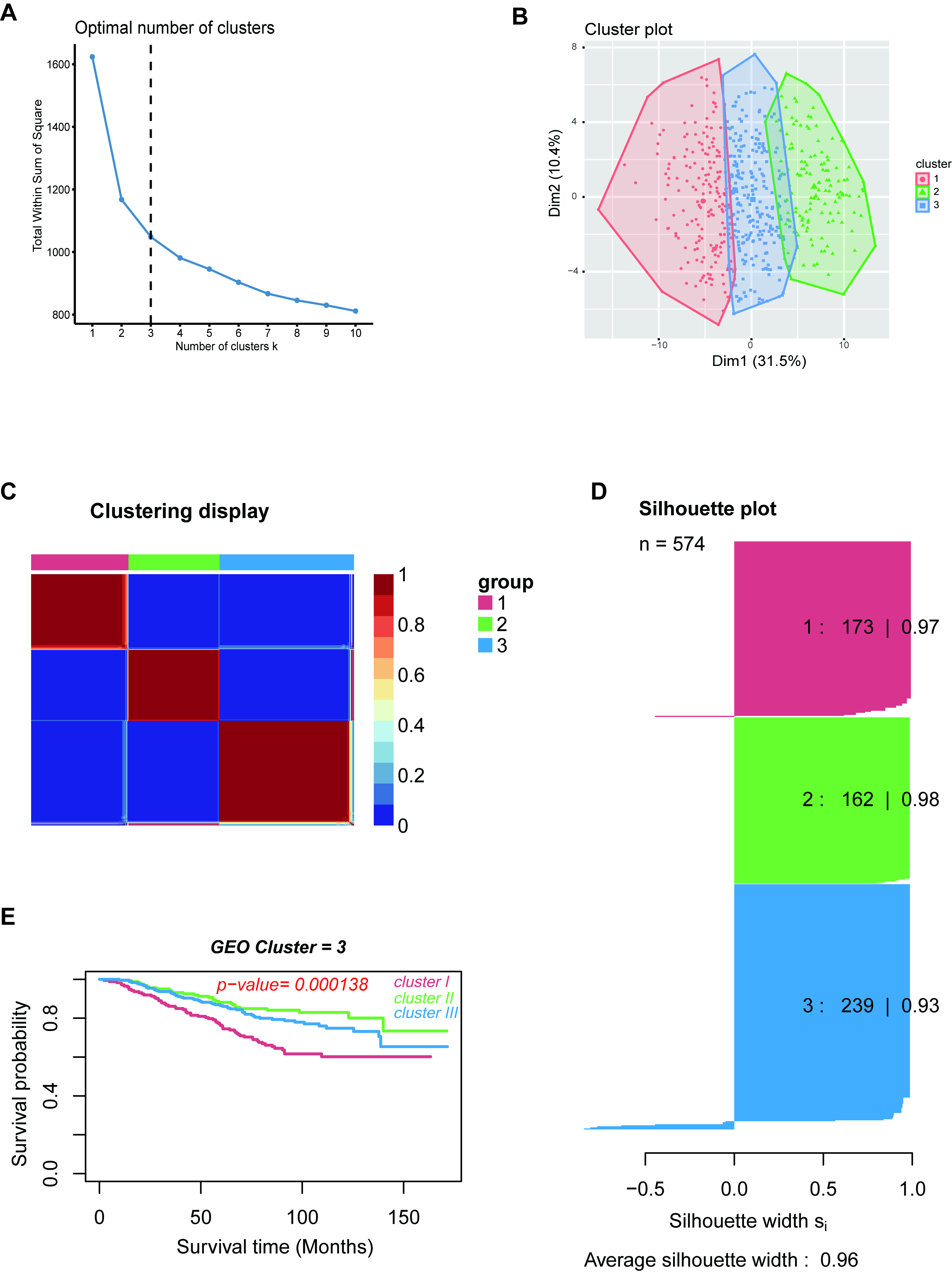
**

**Figure S2. Identification and validation of breast cancer immune subtypes in the GEO cohort.** (A) Selection of the optimal cluster number (k=3) based on the within-cluster sum of squares (WSS). (B) Cluster visualization of BRCA samples projected onto the first two dimensions. (C) NMF consensus clustering heatmap showing the stability of the three subtypes. (D) Silhouette width plot (n=574) showing an average width of 0.96. (E) Kaplan-Meier curves showing distinct survival outcomes among the three subtypes (P = 1.38 × 10^-4^).

**
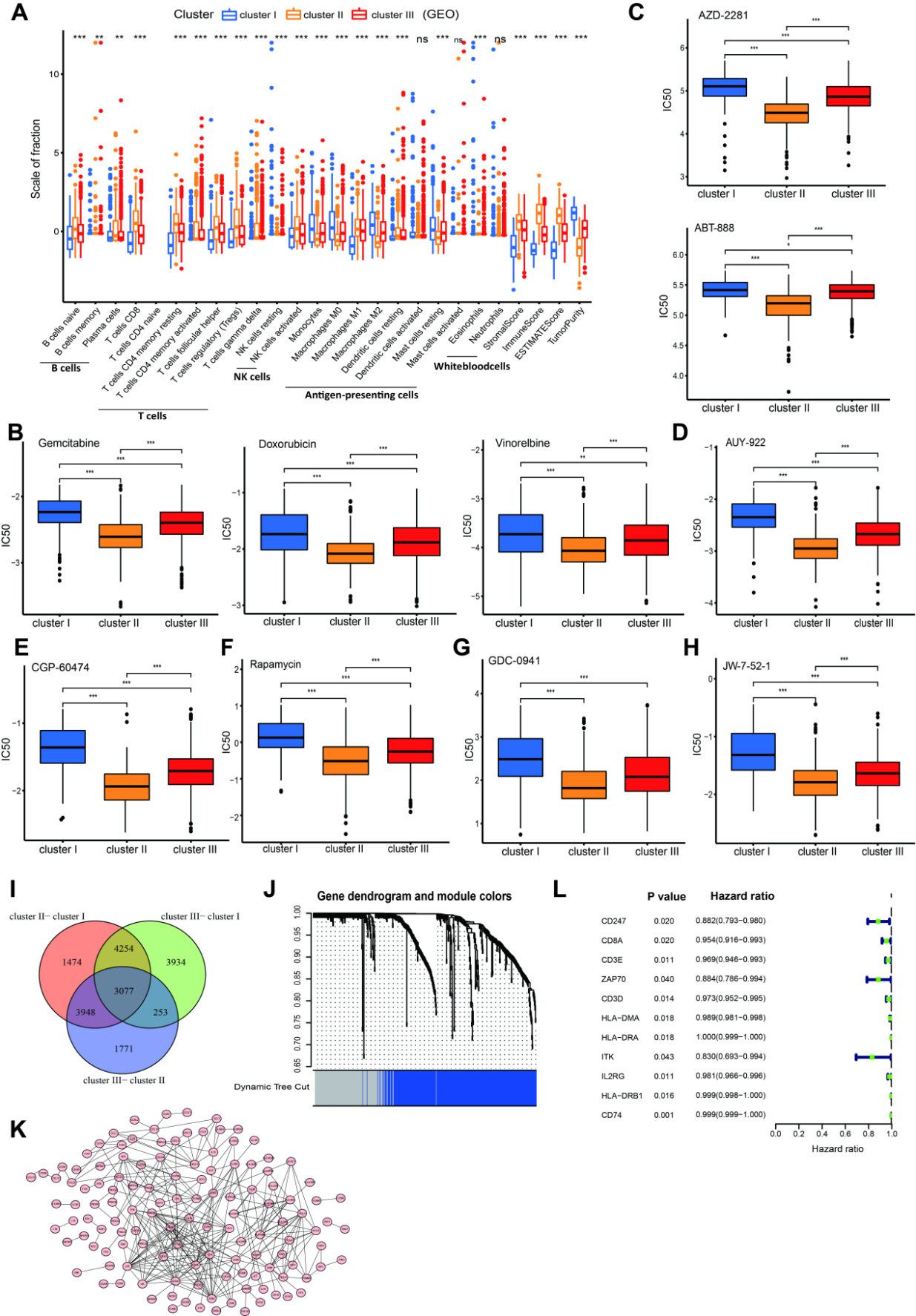
**

**Figure S3.** (A) Distribution of immune cell fractions, microenvironment scores (Immune/Stromal), and tumor purity across the three clusters in the GEO cohort. (B-H) Predicted IC50 values for chemotherapeutic agents—including Gemcitabine, Doxorubicin, and Vinorelbine, and targeted therapeutics —including AZD-2281, ABT-888, AUY-922, Rapamycin, GDC-0941, and JW-7-25-1; Cluster II shows lower values (higher sensitivity). (I) Screening of immune-related genes from differentially expressed genes (DEGs) for WGCNA. (J) Identification of co-expression gene modules by WGCNA. (K) Cytoscape visualization of the filtered interaction network (score > 0.95) and its hub genes. (L) Stepwise screening identifies five pivotal hub genes based on prognostic significance and differential expression.

**
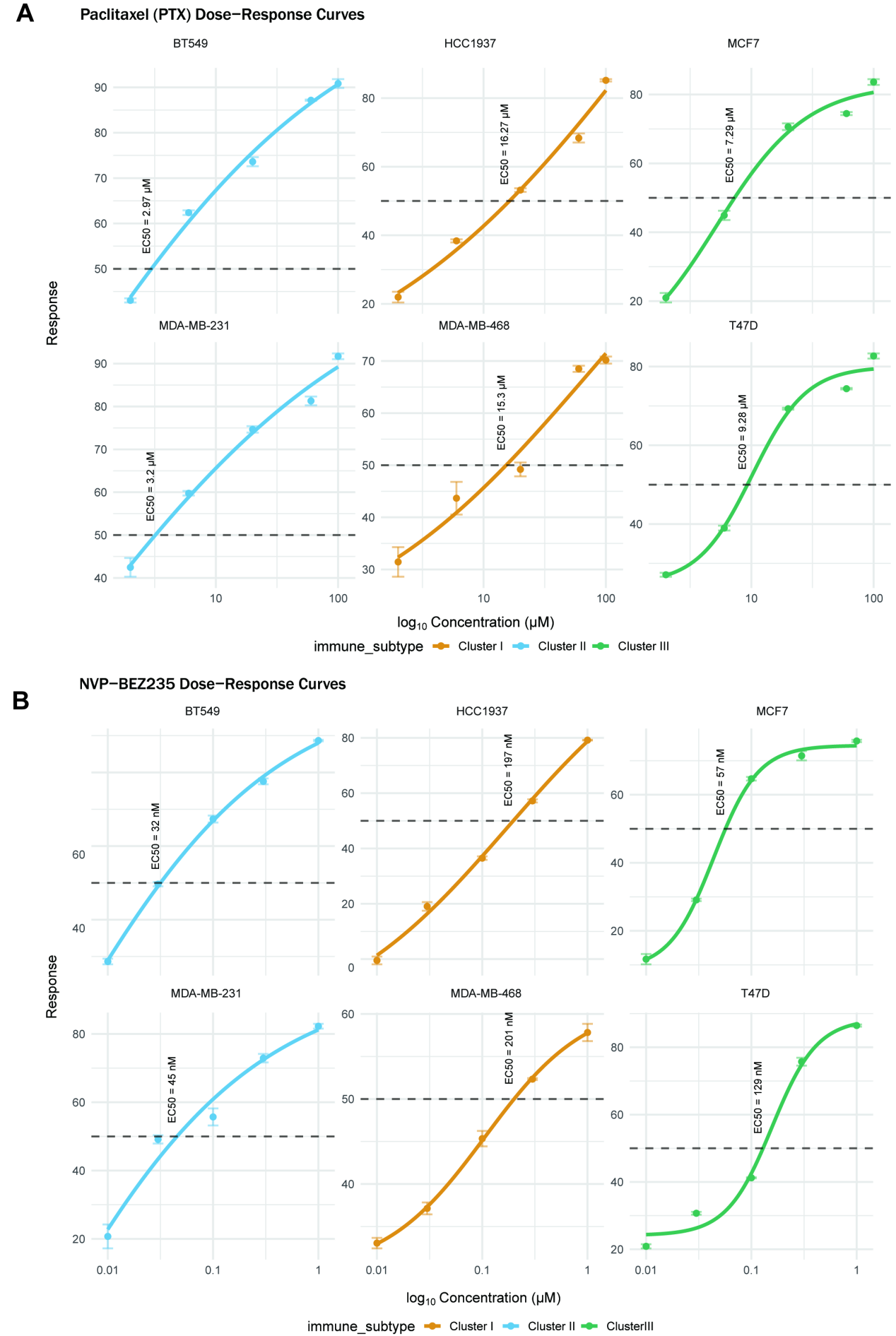
**

**Figure S4. Dose‑response curves of Paclitaxel and NVP‑BEZ235 in breast cancer cell lines representing three immune subtypes.** (A) Paclitaxel (PTX) and (B) NVP-BEZ235 dose-response curves for six breast cancer cell lines stratified by immune subtype (Cluster I: orange, Cluster II: blue, Cluster III: green). EC₅₀ values are labeled for each cell line, with dashed lines marking the 50% response threshold.
